# Getting health back on the highest political agenda—the UN High-level Meetings on health in 2023

**DOI:** 10.1016/S2214-109X(23)00166-3

**Published:** 2023-03-28

**Authors:** Svetlana Akselrod, Gabriela Cuevas Barron, Katie Dain, Lucica Ditiu, Helga Fogstad, Corine Karema, Justin Koonin, Akihito Watabe

**Affiliations:** a Global Noncommunicable Diseases Platform, Geneva 1211, Switzerland; b UHC2030, Geneva 1211, Switzerland; c Partnership for Maternal, Newborn and Child Health, Geneva 1211, Switzerland; d NCD Alliance, Geneva 1202, Switzerland; e Stop TB Partnership, Geneva 1218, Switzerland; f RMB Partnership to End Malaria, Geneva 1218, Switzerland

In September, 2023, world leaders will have a unique opportunity to commit to bolstering health systems, to end tuberculosis, and to invigorate progress on health for all, as three United Nations General Assembly (UNGA) High-level Meetings (HLMs) on health will take place back-to-back in New York, USA. The HLMs on pandemic prevention, preparedness, and response (PPR); universal health coverage (UHC); and tuberculosis, on Sept 20–22, can help to place health high on the political agenda. This attention is urgently needed, because most health-related targets in the Sustainable Development Goals (SDGs),^1^ including those on pandemic PPR, UHC, and tuberculosis, have been thrown off track and inequalities have widened following the COVID-19 pandemic. Increased conflicts and climate change threats have left countries and vulnerable populations at even greater risk in the face of emerging health challenges and social justice.

However, this period of multiple, complex crises also offers a rare opportunity to work together and use lessons learned from the pandemic. We can rally world leaders to make actionable commitments backed by investments in equitable, resilient, and sustainable health systems. We can use these three HLMs to strengthen synergies between health-related SDGs and support governments to effectively prioritise health needs to address multiple challenges such as rising disease burdens caused by non-communicable diseases and mental health conditions, and limited progress on ongoing response to communicable diseases. We have an opportunity to ensure that millions of people, young and old, especially women, children, and adolescents, can access the quality and affordable preventative, diagnostic, and therapeutic services they need.

In 2021, we came together as the Coalition of Partnerships for UHC and Global Health^2^ to unite behind a common goal to align advocacy and accountability efforts to achieve UHC and other health-related SDGs. Now more than ever, we stand behind this goal and our collective efforts.

Strengthening integration across the levels of care and life course, and expanding access to health services that are truly people-centred, have proven challenging in countries. In response, national health plans need to be better at reflecting on priority actions for health systems and integrated approaches with adequate budgets, effective implementation, and financing. Despite continued increases in overall health expenditure during the COVID-19 pandemic,^3^ many essential health interventions remained unfunded. Many countries must prioritise allocating sufficient funding for essential health packages.^4^

With the three HLMs this year, world leaders can commit to more efficient global health architecture, closer and accountable partnerships, and transformative actions for ensuring equitable, resilient, and sustainable health systems so that they deliver for all people in periods of crisis and calm. Countries with people-centred, equity-focused health systems with strong primary health care and community participation are more resilient, sustainable, and better prepared to respond to crises, including maintaining access to essential services under emergency conditions.^5^ Governments must provide financial protection to keep out-of-pocket payments minimal so vulnerable populations can continue accessing the care and services they need at all times. We know issues affecting the access and uptake of health services, such as migration, natural disasters, and climate change require solidarity across countries. We also know that when effective policies inclusively respond to people's needs, we can truly address them. Identifying, mitigating, and overcoming human rights and gender-related barriers is paramount if we want to achieve health for all.

In March, 2023, the UHC Movement launched an Action Agenda,^6^ recognising that all countries require more ambitious, concrete actions and milestones to advance progress on UHC. Political commitment to date has been substantial, but more is needed to get countries on track to deliver on their targets. Recognising that strengthening health systems, ending epidemics such as tuberculosis and malaria, and ensuring pandemic PPR go hand in hand, the Action Agenda will inform the political declaration on UHC and contribute to the other health-related declarations.

As the Coalition, we urge all leaders to unite behind this coherent, integrated health agenda. Specifically, the Action Agenda provides a blueprint for areas that cannot be neglected, such as the health workforce, financing, and gender equality, as well as ensuring that health security is embedded in health systems.

This September, the three UNGA HLMs on health and the SDG Summit provide us with an opportunity to reflect on the progress made and gaps in following the Global Health Commitments adopted by previous UNGAs since 2015,^7^ strengthen collective accountability, and commit to truly transformative changes towards 2030.

For health to be prioritised by heads of state at all times, we call for health progress review summits every 2 years at the UNGAs in 2025, 2027, and 2029, in addition to the specific HLMs, to enhance coherence and leverage synergies among existing processes with all health-related SDGs under a common goal—ensuring health for all to become a reality for everyone, everywhere.

We declare no competing interests.
